# Local circulation of elites punctuated by transregional mobility enabled steppe political consolidation in the Xiongnu nomadic state

**DOI:** 10.1371/journal.pone.0298593

**Published:** 2024-04-01

**Authors:** Cheryl A. Makarewicz, Christine Winter-Schuh, Meghan Jackson, Erik G. Johannesson, Chunag Amartuvshin, William Honeychurch

**Affiliations:** 1 Archaeology Stable Isotope Laboratory, University of Kiel, Kiel, Germany; 2 Institute for Prehistoric and Protohistoric Archaeology, University of Kiel, Kiel, Germany; 3 Meghan Jackson, Ossifrage Exploration Consulting LLC, Huelva, Spain; 4 Erik G. Johannesson, Circle CRM Group Inc., Calgary, Canada; 5 Chunag Amartuvshin, Archaeological Research Center, National University of Mongolia, Ulaanbaatar, Mongolia; 6 William Honeychurch, Department of Anthropology, Yale University, New Haven, CT, United States of America; University of Padova: Universita degli Studi di Padova, ITALY

## Abstract

The Xiongnu polity (ca. 200 BC– 150 AD) emerged out of indigenous community-centered socio-political structures to forge a powerful state that commanded the Mongolian steppe and beyond. Underpinned by a highly mobile pastoralist population, accustomed to seasonally rhythmic moves and embedded in an equestrian culture that facilitated rapid transport over long-distances, it remains unclear precisely how the movement of commoners, local aristocrats and regional elites abetted the formation and organization of Xiongnu state structures. Here, we evaluate Xiongnu movement and dietary intake through multi-stable isotopic analyses of tooth enamel from directly dated Xiongnu intermediate elites recovered from the mortuary center of Baga Gazaryn Chuluu–a prominent granite outcrop set in the Gobi Desert. Carbon isotope (δ^13^C) analysis indicates millet was consumed by some individuals, but whether or not this C_4_ cultivar contributed to the diets of most elites remains ambiguous in this C_3_/C_4_ desert-steppe environment. The effectiveness of oxygen isotopes (δ^18^O) to establish mobility appears much reduced in steppe environments, where geospatially sensitive information appears disrupted by extraordinary seasonality in meteoric water oxygen isotopes, pronounced oxygen isotopic variation in potential drinking water sources, and culturally mediated drinking practices. Most revealing, strontium isotopes (^87^Sr/^86^Sr) indicate circulation of local elites around this central place and beyond, a mobility format that helped leaders cement their own position through political consolidation of spatially dispersed mobile pastoralist communities. The consistent presence at Baga Gazaryn Chuluu of extra-local intermediate elites also points toward the importance of transregional mobility in binding together the Xiongnu polity over the vast distances of the eastern steppe.

## Introduction

The ancient scribes composing historical texts on the Central Plain of China referred to nomadic polities of the Mongolian steppe to the north as “states on horseback” for a very specific reason. Long-distance and rapid mobility based on expertise in horsemanship was built into the cultural, social, and political systems of these steppe populations [1]. Politics and mobility went hand-in-hand to configure extensive regional polities with high levels of interaction despite low population densities commensurate with most pastoral nomadic societies, ultimately forming large-scale and militarily powerful political organizations that have been referred to in the scholarly literature as confederations, empires, and most recently as Inner Asian “Great States” [2]. A key question about these so-called nomadic polities is how traditional forms of seasonal movement and long-range mobility, guided by broader socio-political forces, might have played key roles in the process of regional integration that supported state formation [3]. Despite numerous studies hypothesizing that steppe politics were built upon a culture of movement, how mobility formatted state development on the steppe has yet to be established.

**Fig 1:**
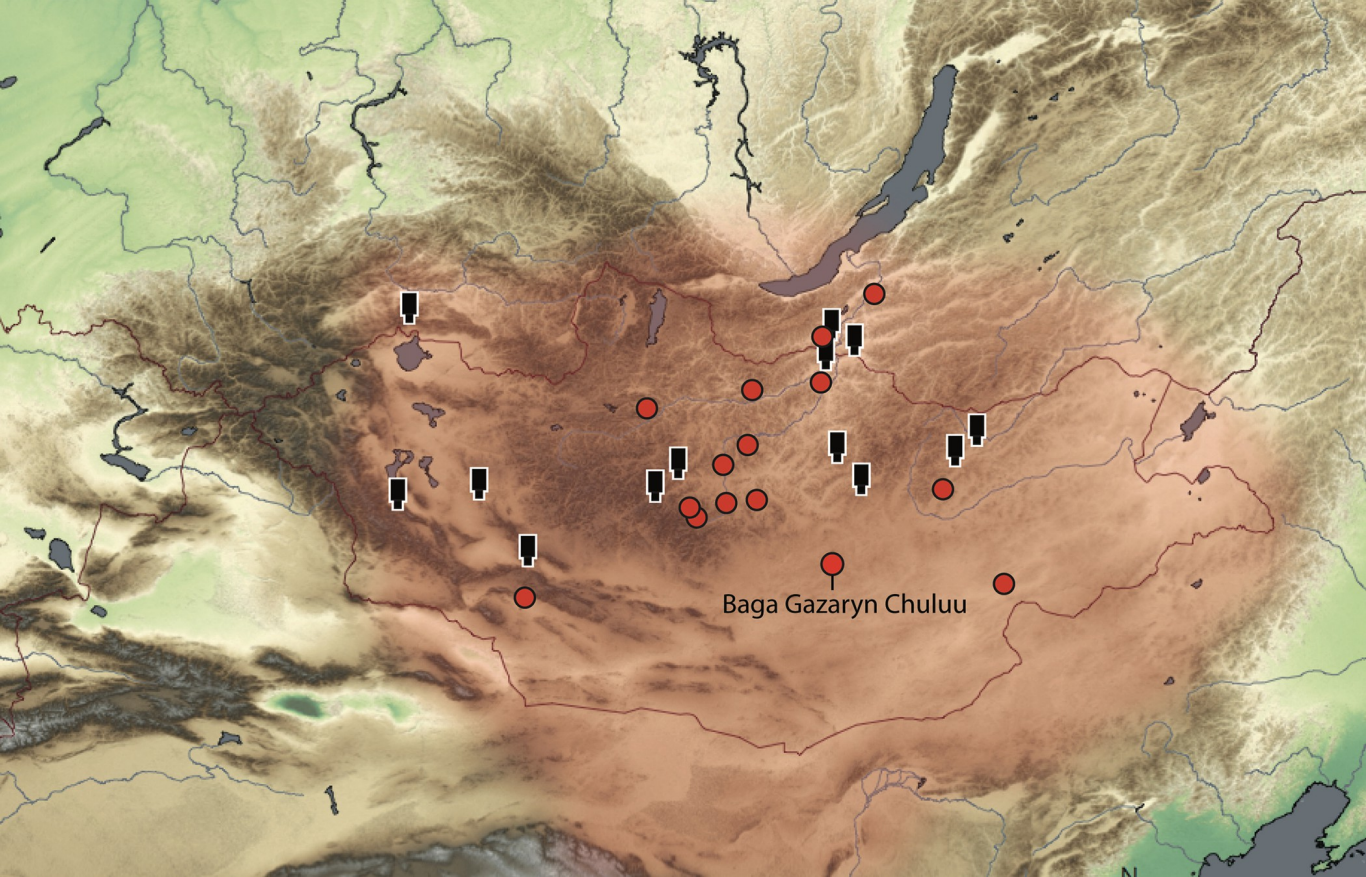
The extent of the Xiongnu Empire (ca 250 BCE– 150 AD) indicated in red and the location of Baga Gazaryn Chuluu, a Xiongnu intermediate elite ring burial cemetery located in the Gobi desert steppe, Mongolia. Red circles indicate major ring burial cemeteries supporting 100+ burials. Black icons indicate distribution of aristocratic elite terrace tombs.

The Xiongnu polity (3^rd^ c. BC − 2^nd^ c. AD) was the first of these nomadic states to emerge upon the Mongolian steppes as a contemporary of the Qin and Han dynasties seated in China (Fig 1).

This regionally integrated polity arose from indigenous, steppe-centered socio-political developments drawing on earlier Late Bronze and Early Iron Age (ca. 1200 − 300 BC) forms of social organization emphasizing community cohesion and inter-community social interaction [3, 4]. The Xiongnu state was supported by internal pastoral production, a degree of cereal crop agriculture that likely supplied local and regional elites, and the management of long-distance exchange networks and tribute extraction [5]. Distinguished from other Old World states populated by sedentary agriculturalists and directed by centralized and urban administrations, the Xiongnu polity was underpinned by a large population practicing mobile herding and multi-seasonal movements of households. This traditional mobility was further enhanced by an equestrian culture that facilitated rapid movement and communication over long distances [5].

How pastoralist mobility may have underwritten political motives that sought to promote internal integration within an emerging Xiongnu state structure remains unknown. Pastoral movement appears to have been constrained by spatially bounded polities during the later Early Iron Age (ca. 500–300 BC), but regional integration during the subsequent Xiongnu period opened opportunities for increased mobility and more expansive pastoral movements that may have been important for supporting a burgeoning extra-local pastoral economy [6]. Local elites and their households would have actively circulated amongst extra-local neighboring communities to build political support through in-person alliance relationships, gift-giving, and inter-marriage [3]. Such politically motivated movement would have been fundamental to political faction building that eventually contributed to the making of sub-regional “aristocratic houses” composed of local, intermediate elites that supported the Xiongnu polity [7].

While historians refer frequently to the habitual speed and mobility of steppe life, the challenge for archaeologists is to document the occurrence, frequency, and extent of pastoralist mobility. Recent analytical advances have opened up new opportunities to study a wide range of pastoralist mobilities through examination of technologies, habitation site arrangements, landscapes, genetics, and perhaps most significantly, through isotopic analyses of skeletal remains [8–10]. Strontium, oxygen, and carbon isotopes in calcified tissues record environmental inputs that help track ancient human mobility although not without significant questions, emergent problems, and ongoing debate to refine analytical methodologies [11]. Given the embedded nature of movement in pastoral nomadic societies, especially among groups with equestrian traditions, these isotopic approaches would seem a good fit for the archaeologist working on mobile herding communities. So far, however, the application of isotope analyses to identify human and animal movements in Mongolia have been relatively few and far between [12–14].

Here, we investigate the movement histories of intermediary elites embedded in mobile pastoral communities during the four-century span of what might be called the first Great State of Mongolia − the Xiongnu polity. In particular, we assess ebbs and flows in regional and trans-regional elite mobility to evaluate how movement configured the organization of Xiongnu state structures. We examine Xiongnu movement through strontium and oxygen isotopic analyses of well-dated human skeletal remains recovered from cemetery sites located at Baga Gazaryn Chuluu (BGC), a central locale for Xiongnu monument construction and mortuary activity (Fig 1). Our analysis comprises three steps. First, we define bioavailable strontium isotope values for the BGC region through analyses of contemporary and Xiongnu period domesticated livestock and a comparative sample of domesticated and wild sheep native to the area. We then compare and critique the suitability of strontium and oxygen isotopes as a means to trace human movement on the Mongolian steppe. Finally, we document the carbon isotopic composition of tooth enamel carbonates in order to establish the role of millet in Gobi Desert Xiongnu period diets. In doing so, we determine that the manner in which households moved and what they chose to eat were important components for constructing Xiongnu statehood.

## The Xiongnu archaeological record

The territorial extent of the Xiongnu state is not clearly understood, but at its zenith the polity controlled or exerted influence over much of the eastern Eurasian steppe zone. In addition to a limited number of historical sources from the neighboring Han Dynasty, our knowledge of Xiongnu socio-political organization and interaction is based primarily on archaeological research from Mongolia, the Transbaikal region South Siberia and Inner Mongolia [15]. Systematic survey and reconnaissance document the use during the Xiongnu period of both large walled settlements and small seasonal campsites. Attention to these habitations is increasing but to date, only a relatively small number of settlement sites have been studied [16]. Walled settlements seem to have been used as palace or ceremonial sites for the uppermost elites [17], while smaller settlements housing domestic semi-subterranean dwellings, workshop areas, and architectural structures attest to quotidian occupation by commoners [18].

Seasonally occupied nomadic campsites used by Xiongnu herders are usually represented by a central hearth feature surrounded by sparse artifact scatters with minimal debris accumulation and no detectable architectural investment. This material patterning is consistent with the use of portable dwellings (i.e., yurts or gers) depicted in images on a number of Xiongnu birch bark containers [16, 19]. Seasonal habitat sites also yield the scattered remains of domesticated sheep, goat, cows, horse, and, occasionally, dogs [20, 21], while stable isotopic evidence indicates that herd animals were seasonally foddered and pastured during the winter season [12]. Wheat, barely, and millet recovered from burial and habitation contexts suggest grain cultivars were consumed by some individuals [22].

While habitation sites and walled settlements are contributing new and insightful information about the Xiongnu period, it is the mortuary record that has received the most attention from archaeologists. These contexts have provided the majority of information on Xiongnu socio-political organization including interregional interaction, social stratification, and differences in resource access and dietary intake. Xiongnu period mortuary monuments are represented by two main forms: large platform (or terraced) monuments and smaller ring burials. Both forms were distributed across Mongolia and southern Siberia (Fig 1), typically identified as the core of the Xiongnu state, and ring burial cemeteries are also known from Inner Mongolia [23]. Each of these monument types involves significant stone surface constructions overlaying deep burials pits and both are fairly consistent in format and furnishings given the geographical and temporal ranges they encompass [15]. These monuments are typically incorporated into cemeteries of varying sizes ranging from a few graves to sites holding hundreds of burials. While large platform burials were treatments reserved for the uppermost elite reflected in their outstanding size, labor investment, and burials furnishings, ring burials served ‘intermediate elites’ and their close lineage groups, indicated by their ancient nuclear and mitochondrial genomes [24, 25], and are the mortuary types pertinent to this study.

**Fig 2:**
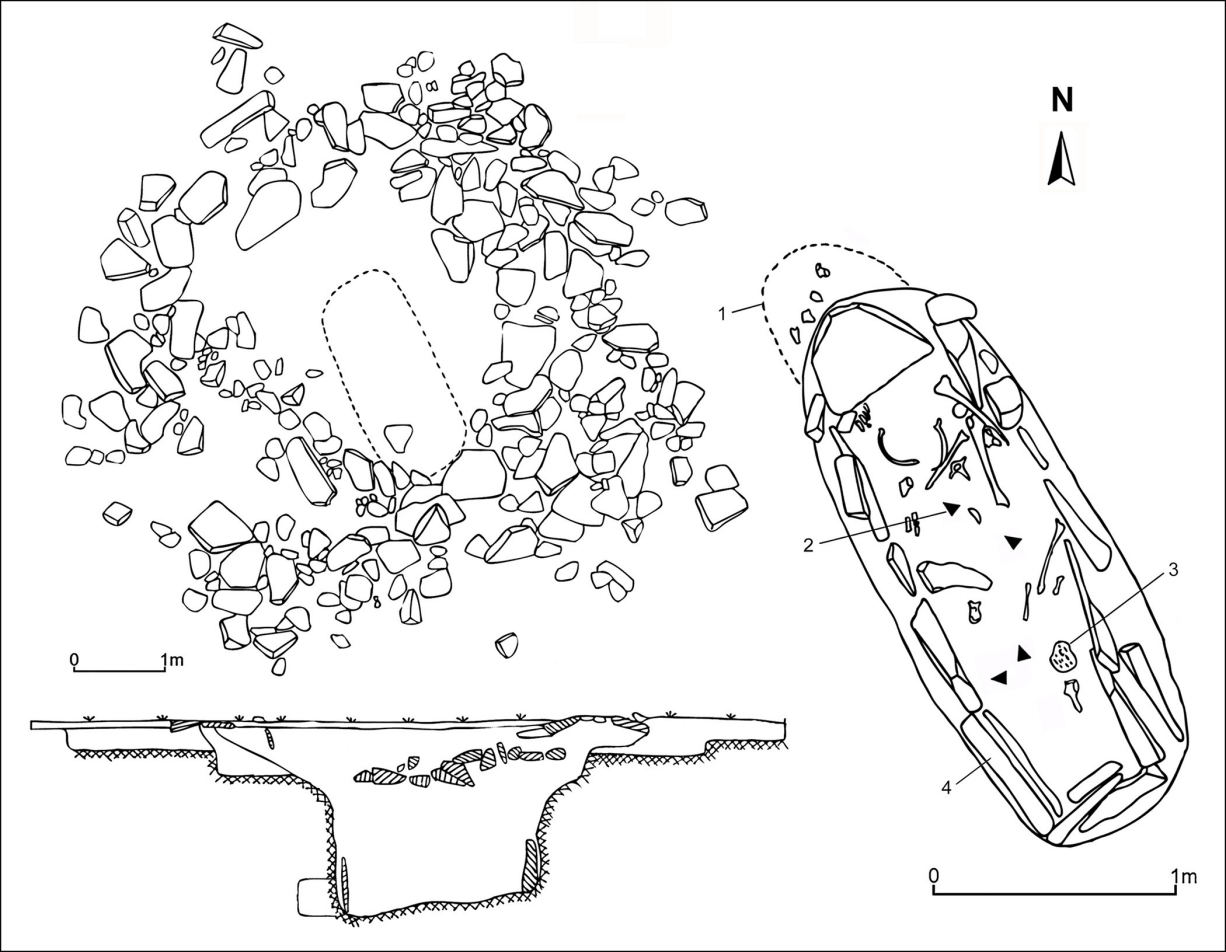
Example of a Xiongnu intermediate elite burial. Burial Ex08.04b containing the disrupted skeletal remains of a male individual, 20–40 years in age. Burial chamber and artifacts include: (1) ceremonial alcove containing herd animal offerings; (2) iron arrowheads; (3) item made of birch bark; (4) stones comprising the internal cist structure of the burial chamber. Ex08.04 also included a later intrusive interment not shown here.

Ring burials consist of large circular embanked surface features constructed of stone and soil that cover earthen or slab-lined burial pits approximately 1 to 4 m in depth [26] (Fig 2). These burials typically contain diverse funerary paraphernalia including elaborate decorated wooden coffins, ceramic vessels, bone tools, iron weapons, horse riding gear, and a variety of long-distance and prestige objects including Han Dynasty lacquer ware bowls and bronze mirrors, glass and stone beads from Central Asia, and expertly worked gold, silver and bronze objects [27]. The remains of sacrificed livestock, in particular the skulls, cervical vertebrae, and the foot parts of sheep and goats but also cattle and horses, were commonly interred in ring burials [20]. Considerable variation in the type and quantity of sacrificial remains suggests distinction in animal-based wealth between local elites or perhaps differences in their ability to garner animal resources from local communities [20].

Correspondence between ring burial construction size and complexity, burial furnishings, livestock offerings, and varieties of exotic prestige goods placed in such burials across Xiongnu territory demonstrates the importance of adherence to formalized sumptuary codes most probably related to social ranking [3, 28, 29]. In addition, materials such as larch timbers for coffins and birch bark goods from forested areas of the steppe regularly appear in the Xiongnu burials of the Gobi Desert and suggest that intra-steppe relationships and exchange were an important part of Xiongnu organization. Based on these observations, funerary activities may have drawn together dispersed mobile communities through ceremonial events that highlighted the political relationships between local leaders and commoners via the shared symbolism embodied in state sanctioned mortuary practice [15]. Nascent Xiongnu local leaders sought to establish authority and legitimize their leadership through these funerary regimes, the acquisition and use of prestige goods, and by participation in trans-regional political networks and alliances.

## Baga Gazaryn Chuluu: The monumental landscape of a gobi desert central place

Baga Gazaryn Chuluu (BGC) is an expansive granite ridge system situated in the arid steppe region of the northern Gobi Desert of Mongolia (Fig 1). A visually arresting geological feature, BGC has served as a major focal point of mundane and ceremonial pastoralist activity for thousands of years. The pastures surrounding BGC supply livestock herds with abundant graze during the summer months while the granites capture and store summer rainfall that later flows as springs. During the cold winter months the rocky interior provides well-protected campsites for herders and their animals along with pasture reserves [30]. Systematic pedestrian survey at BGC has documented more than 1700 archaeological sites, the majority of which were mortuary and monumental constructions, rock art sites and artifact scatters from the Neolithic onwards, demonstrating the importance of this area as an enduring locus for pastoralist as well as hunter-gatherer subsistence and mortuary activities [31].

Eleven major Xiongnu cemeteries ranging from 5 to 75 burials, as well as several single and double Xiongnu burial sites, all located on the margins circling the granite ridges, have been identified at BGC [27]. Although Xiongnu period mortuary sites are qualitatively different from earlier funerary monuments, they are often located in locales associated with earlier Late Bronze and Early Iron Age monumental burials, which suggests both a degree of continuity in landscape use and the prominence of BGC as a focal point in the greater region for funerary activity [32]. Likewise, just as BGC was the primary center in the greater region for Late Bronze and Early Iron Age monument construction (late 2^nd^ to mid–1^st^ millennium BC), Xiongnu period practices continued to focus on BGC as a central place. Indeed, BGC seems to be the major center for Xiongnu activities in this region [3]. Given its prominence within the greater landscape, funerary and ceremonial activities conducted at BGC may very well have contributed to the broader sociopolitical integration of this subregion within the Xiongnu state.

**Fig 3:**
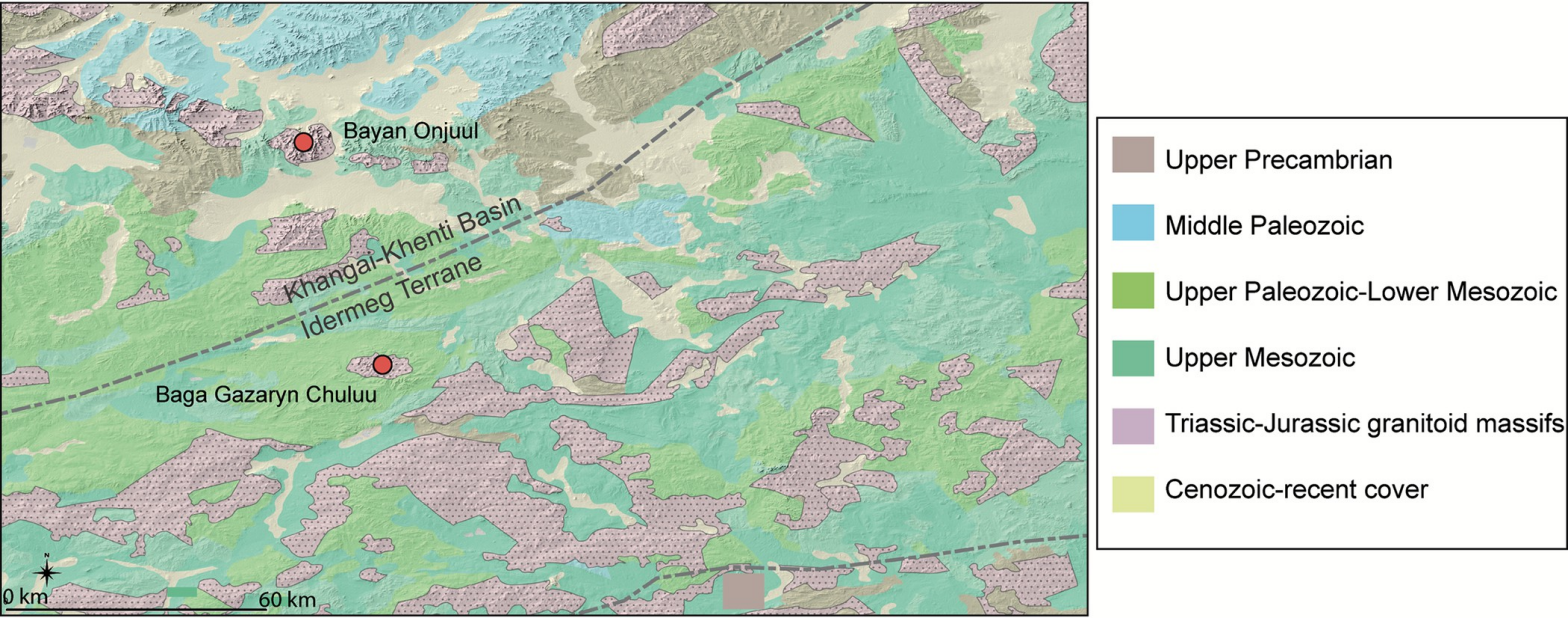
Geology of the greater Baga Gazaryn Chuluu showing the relationship between the volcanic sedimentary country rocks and the granitoid massifs. The granites are of similar but not identical composition due to the terrane boundary between the two areas [33, 34].

Outward from BGC, the next closest major Xiongnu burial ground is located approximately 80 km northwards in an area known as Zorgol Khairkhan in the district of Bayan Onjuul (Fig 3).

Like BGC, Zorgol Khairkhan is a large granite ridge system comprising a substantial number of monumental sites from earlier periods but has relatively few Xiongnu burials in comparison to BGC. As such, Zorgol Khairkhan was likely a secondary center to BGC during the Xiongnu period. Today, herding households maintain close ties between the two locales taking advantage of the ecotone between classic steppe and desert steppe environments. Households with large herds and available transport commonly move their flocks to the Bayan Onjuul region in summer time demonstrating an ecological connection between these areas beneficial to the herding economy [3].

## Stable isotope background

### Strontium isotopes and geology

Strontium isotopes recorded in tooth enamel provide information on the scales of human movement [35, 36]. Geospatial variation in bioavailable strontium isotopes is linked to the strontium isotopic ratio (^87^Sr/^86^Sr) of the underlying bedrock [35, 37–39] and is further influenced by local soil formation processes, glacial action, and the addition of alluvial deposits or aeolian sediments to local soil substrates [35, 40]. Strontium isotopes become biologically available through weathering processes and fractionate little as they enter the foodweb due to their heavy mass [36, 38, 41]. Provided there is enough variability in the strontium isotopic composition of the geological settings and bioavailable strontium isotopes, ^87^Sr/^86^Sr values of skeletal tissues can inform on human movement in ancient landscapes.

The distribution of bioavailable strontium isotope values across the Mongolian steppe is largely unknown but expected to vary widely based on diverse geologies, substantial aeolian cover, and alteration to primary host rock by hydrothermal activity. In southern Mongolia, geologic terrane boundaries run northeast to southwest, juxtaposing island arc and basinal terranes, which were subsequently intruded by post-accretion granitoids of late Triassic to possibly Cretaceous age (Fig 3). These granitoid bodies are prominent both geologically and topographically at both BGC and at Bayan Onjuul [42, 43]. At BGC, the exposed granitoid (236–200 Ma [44]) intrudes into surrounding country rock composed of Permo-Triassic volcano-sedimentary rocks (ignimbrites and sandstones) that form the Idermeg volcanic arc terrane [42, 45]. At Bayan Onjuul, the Jurassic, or possibly younger (121 Ma [45]), granite body intrudes country rocks of the Khangai-Khentii accreted basin terrane composed of Triassic Tumengol volcanic rocks and Devonian Dov volcano-sedimentary sequence, with Cretaceous post-intrusive gravel and sandstone cover (Fig 3) [43].

The two terranes are divided by the Mid-Mongolia Tectonic Line, which falls roughly 30 km north of Baga Gazaryn Chuluu, and the Adatsag Ophiolite, which reflects the final closing suture of the Mongol-Okhotsk ocean [46]. Valley fill within the granite massifs would be expected to more closely reflect the bioavailable strontium isotope signature of the country rocks than that of the granites themselves. Overall, although the BGC and Bayan Onjuul granitoids are of similar composition, the differences in the age of each formation and composition of surrounding country rock, hydrothermal history, and terrane setting would result in contrasts in their strontium isotope composition rather than similarities. Because the BGC-Bayan Onjuul region marks an ecotone important for contemporary herding practices, we might expect that ancient pastoral movements would also have occurred across this area. As such, this geological variation between the two subregions establishes a minimum distance to the north over which we might expect movements to produce non-local strontium isotope ratios in human skeletal material [14].

### Oxygen isotopes

Oxygen isotopes are also used to trace human residential mobility in a variety of ancient cultural contexts and environmental settings around the globe [47–49]. Oxygen isotope ratios of mammalian tissues reflect the oxygen isotopic composition of body water which is influenced by the δ^18^O of ingested water, organic compounds in food, and inspired atmospheric oxygen [50–52]. The relative contribution of each source to mammalian body water pools is poorly understood, as is the impact of various metabolic processes on the oxygen isotopic composition of body water incorporated into tissue during synthesis. However, the δ^18^O values of imbibed water appear to most heavily affect the oxygen isotope ratios of tooth enamel carbonates [53]. The δ^18^O of enamel carbonate, which is derived from blood bicarbonate, is directly related to body water δ^18^O due to the rapid enzymatic transfer of oxygen between blood and bicarbonate [54–57]. The oxygen isotopic composition of human tooth enamel is heavily influenced by the δ^18^O value of local drinking water sources, which often, although not always, bears a strong relationship with the oxygen isotopic composition of local meteoric waters which are in turn determined by continental positioning, relative humidity, altitude, temperature, and rainfall amount [58, 59]. The geospatial sensitivity of oxygen isotopes to these environmental factors potentially generates considerable isotopic variation within and between geographic regions, providing a framework to evaluate the movement of individuals across landscapes.

Mongolia is characterized by a substantial latitudinal environmental gradient with cooler temperatures, moderate precipitation levels, and forest steppe present in the north and warmer, more arid conditions present in the Gobi Desert to the south. Reflecting differences in precipitation amounts and temperature, meteoric water is increasingly enriched in ^18^O with a corresponding decrease in latitude across the Mongolian steppe [60, 61]. Regional differences in the oxygen isotopic composition of meteoric water are mirrored in the δ^18^O values of bulk sampled tooth enamel carbonate from obligate drinking horses, which are depleted in ^18^O for animals inhabiting northern latitudes and enriched in ^18^O for horses from the warmer and more arid environments of the south [13, 60].

Strong seasonality in local temperature and amount of precipitation also impart considerable seasonal variation in local meteoric water δ^18^O values. Today, BGC experiences relatively hot summers and extremely cold winters with average temperatures for July and January 19°C and −17.5°C, respectively, and precipitation levels averaging 160 − 200mm per year with most precipitation falling as rain during the mid-to late summer (July–September). The oxygen isotopic composition of meteoric waters collected in 2003 at the Mandalgovi research station, located ca. 50 km south of BGC, shifts on a seasonal basis and ranges from −27‰ to −9.3‰ in δ^18^O_VSMOW_ [61]. This seasonality in environmental oxygen isotopes is reflected in the oxygen isotope ratios of tooth enamel carbonates of herbivores grazing and browsing in the steppe desert region. Sequentially sampled teeth from modern domesticated sheep local to BGC demonstrate a seasonal range in δ^18^O_VPDB_ ranging on average from −15.3 to +1.5 ‰ (Δ^18^O = 16.6‰), while local argali sheep exhibit a more restricted range averaging −11.4 to −1.5‰ (Δ^18^O = 9.9‰). The oxygen isotopic divergence between wild and domesticated caprine taxa reflects differences in the amount of ^18^O-enriched leaf water and ^18^O-depleted groundwater from wells imbibed by domesticated and wild sheep [62].

### Carbon isotopes

In steppe environments, carbon isotopic variation at the floral base of the foodweb is driven by variation in the proportion of C_3_ and C_4_ plants on the landscape averaging −26‰ and −12‰ in δ^13^C, respectively, and also by water availability which influence C_3_ plant carbon isotope ratios [63–66]. Carbon isotope analyses of skeletal tissues document dietary intake, providing a powerful means to estimate the relative importance of foodstuffs with distinct isotope compositions to human diets. One such food that may have contributed to ancient Xiongnu diets is millet, a nutritionally rich C_4_ crop characterized by a high tolerance to poor water availability. Well suited to semi-arid growing conditions, millets require relatively low labor and management inputs for substantial grain yields [67]. Tracing millet consumption, in particular low-level millet intake, in the Mongolian steppe through carbon isotopic analysis of human bone collagen has been confounded by the abundance of ^13^C-enriched plants in steppe environments, which include C_3_ plants seasonally enriched in ^13^C due to low water availability and C_4_ grasses and chenopods present in the desert steppe [68, 69], ingested by livestock that provide pastoralists with protein.

Here, we measured carbon isotope ratios from tooth enamel bioapatite which reflects the carbon isotopic composition of ‘whole’ diet, i.e., proteins, carbohydrates and lipids, in contrast to collagen which reflects only the protein portion of the diet [70, 71]. Structural carbonate in biogenic hydroxyapatite is derived from dissolved inorganic carbon (DIC) synthesized primarily from carbohydrates and lipids with some contribution from dietary protein [72, 73]. In order to facilitate comparison of human carbon isotope values with those measured from local fauna, we applied to human carbonate δ^13^C values a diet-apatite carbonate fractionation factor of δ^13^C_diet_ = (1.04 * δ^13^C_apa_)– 9.2 according to Ambrose and Norr [70].

## Materials and methods

### Bioavailable strontium isotopes in Baga Gazaryn Chuluu

The local bioavailable distribution of strontium isotopes in BGC was established through analysis of enamel samples from both modern and archaeological sheep from the BGC area. Second mandibular (M/2) teeth from three (n = 3) modern domesticated juvenile male sheep (*Ovis aries*), all from the same flock and herded in the BGC area throughout their lifetime, were analyzed. These sheep were regularly moved to various pastures located in the open steppe within a 20-km radius of BGC during the summer months but were relatively stationary during the winter when animals were grazed on reserved pastures associated with winter camps located in the lower valley folds of BGC [30]. Sheep were also provisioned during the winter with fodder collected from summer pastures located within or in the immediate surround of BGC [30, 74]. Archaeological sheep teeth (*n* = 4) recovered from two Xiongnu ring burials, BGC 128 (Ex03.03) and BGC 576 (Ex04.01), were also analyzed here for strontium isotopes. The upper second molars of two modern wild argali sheep (*Ovis ammon*) were also selected for strontium isotopic analyses in order to establish directly the strontium isotopic composition of the granitic pluton. The movement of argali sheep is largely restricted to the granite ridges of BGC that support sparse graze and woody bushes [74].

### Human teeth

**Fig 4:**
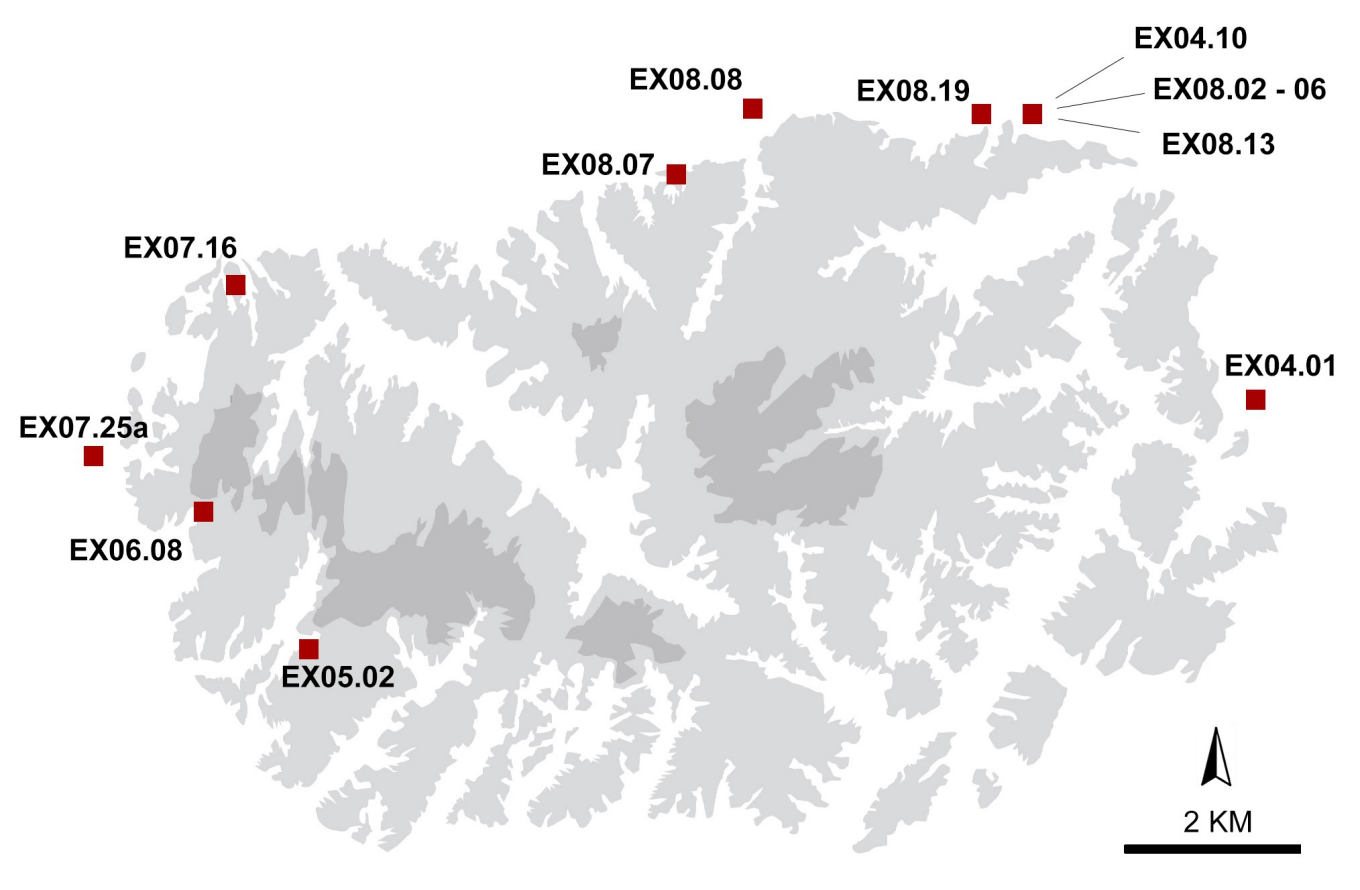
Map of the Baga Gazaryn Chuluu granite ridge system and the locations of Xiongnu period cemeteries where excavations provided samples for isotope analyses. Areas marked in dark gray range in elevation from 1600 to 1770 m a.s.l., light gray indicates 1600 and 1530 m a.s.l., and white represents landscape below 1530 m a.s.l.

A total of 22 teeth from 15 individuals recovered from directly dated Xiongnu ring graves were selected for strontium, oxygen, and carbon isotope analysis. Ring burial contexts excavated during the Baga Gazaryn Chuluu survey and bioarchaeology project (2003 − 2008) provide all the human samples for isotopic analysis reported here (Fig 4). Strontium isotope values published in Machicek et al. (2019) [14] representing seven of the same individuals analyzed as part of this study, but different teeth with different formation times, and also one additional individual, were also incorporated into our study to more closely examine the mobility dynamics of individuals. Teeth do not remodel after formation and, consequently, enamel isotopic composition reflects environmental inputs and dietary intake that took place throughout childhood and juvenile years. Assorted permanent teeth with different formation times, including the canine, incisor, premolar, and molar, were sampled reflecting the availability of tooth remains available for analysis. The teeth sampled as part of this study form at different rates and complete crown formation at different times in the following order: first molar (M1) with crown formation complete between ca 2.5−3 years, the central incisor (I1) and lateral incisor (I2) between ca. 4−5 years, the first premolar (P1), the canine (C) and the second premolar (P2) between ca. 6−7 years and the second molar (M2) between ca. 7−8 years [75]. The mineralization time of the third molar is quite variable, with completion during later adolescence. The tooth specimen assemblage, despite its eclectic character, permits documentation of early life mobility histories of individuals interred in BGC ring burials.

Mineralization of teeth proceeds along cone-shaped appositional fronts with enamel formation taking place over several years [76]. Here, a vertical slice of enamel was cut into the tooth loph from the cusp to the enamel-root junction to catch the total intradental variation of ^87^Sr/^86^Sr. For some teeth it was not possible to sample the complete length of the loph. These subsamples may underestimate the whole strontium isotopic variation of the tooth but still represent long-term averages of ^87^Sr/^86^Sr for early childhood due to the long residence time of strontium in the body and the extended enamel mineralization time [77].

Tooth enamel is highly robust against diagenetic alteration due to the high crystalline structure and low organic matter of enamel hydroxyapatite [78]. Although the isotopic composition of metabolically dynamic bone reflects a ‘lifetime’ average of dietary intake, providing information on the movements individuals undertook later in life, bone was excluded from this study. Bone apatite is highly susceptible to recrystallization once deposited in the burial environment due to high organic content, high porosity, and poor crystalline structure of the bone [79, 80]. Dissolution and recrystallization of bone apatite perturb biogenic strontium and oxygen isotope values [81], while the approximately 1.4‰ ±1.0 offset between tooth and bone apatite carbonate δ^18^O values and consistent ^18^O enrichment in enamel values relative to bone carbonate values impedes direct comparison of isotope values derived from bone and teeth [82].

This study does not convert δ^18^O_apa_ values into corresponding drinking water oxygen isotope values (δ^18^O_DW_) for direct comparison with geospatially sensitive meteoric water values (δ^18^O_MW_) in order to reconstruct mobility or geographic origins of human individuals. Although this approach has been used in other mobility studies, recent work has demonstrated that enamel conversion equations compute disparate δ^18^O_DW_ values [83, 84]. We also do not directly compare human δ^18^O_carb_ values to those exhibited by modern or contemporaneous Iron Age sheep from BGC for two reasons. First, the oxygen isotopic composition of domesticated sheep is directed, in addition to meteoric water inputs, by the oxygen isotopic composition of leaf water that is enriched in ^18^O relative to meteoric water [62]. Although the amplitude of oxygen isotopic differences between semi-obligate drinkers such as sheep and obligate drinking humans is not yet established and is expected to vary widely depending on leaf water intake, sheep from the same geographic locale as humans are expected to exhibit higher δ^18^O values. Second, mean δ^18^O values obtained from sequentially sampled teeth, as was the case for BGC sheep, are typically offset relative to the δ^18^O values of a bulk sample obtained from the same sampled tooth, hindering direct comparisons [85].

### Sample preparation and measurement

Individual tooth specimens were sub-sampled with at least half of the tooth remaining in its original position situated within the maxillary or mandibular dental arcade, elements which are stored at the Mongolian Institute for Archaeology. Any remnant specimen sub-sample not consumed for isotope analyses are stored in the Archaeology Stable Isotope Laboratory, Kiel, Germany. All necessary export permits were obtained for this study, secured by Dr. Chunag Amartuvshin in 2008 from the Culture and Arts Office of the Ministry, Culture and Science, Ulaanbaatar, Mongolia, which complied with the relevant regulations. Tooth crown surfaces were abraded using a diamond tipped drill in order to remove adhering sediments. For strontium isotope analysis of human teeth, enamel slices were removed from the tooth crown with a circular diamond edged dental saw (see S1 Table for additional sampling details). For sheep, strontium samples were taken from points on the tooth crown that coincided with the highest δ^18^O values representing a summer period of tooth mineralization. Radiogenic strontium isotope analysis of tooth enamel samples was conducted at the clean laboratory of the GEOMAR Helmholtz Centre for Ocean Research at Kiel. Samples were weighed into clean Teflon beakers and dissolved in 8 M HNO_3_ and H_2_O_2_, evaporated to dryness, redissolved in 8 M HNO_3_ and finally loaded on chromatographic columns filled with Eichrom strontium-specific resin. Strontium was separated from the sample matrix by washing the column with 8 M HNO_3_ and eluted using 0.05 M HNO_3_. After separation, the solutions were dried down, followed by heating with a mixture of concentrated HNO_3_ and H_2_O_2_. Strontium concentrations, measured before strontium separation, were analyzed using Quadrupole-Inductively Coupled Plasma-Mass Spectrometry (Agilent 7500cx). ^87^Sr/^86^Sr ratios were measured in a Neptune MC-ICP-MS. Measurements of the NBS 987 standard yielded an average ^87^Sr/^86^Sr value of 0.710209 ± 0.000006 (1σ, n = 4). Radiogenic ^87^Sr/^86^Sr ratios of the samples were normalized to a ^86^Sr/^88^Sr ratio of 0.1194 [23, 38]. Samples were also corrected for the offset between the measured ^87^Sr/^86^Sr value of SRM987 of the individual session and the ^87^Sr/^86^Sr ratio of 0.710240 as published in Veizer et al. [86].

Stable oxygen (δ^18^O) and carbon (δ^13^C) isotopes were measured from the structural carbonate fraction of enamel bioapatite. Bulk powdered enamel sample was removed from the tooth crown using a Dremel drill equipped with a diamond drill tip. Powdered enamel samples were treated with 0.1 M acetic acid (0.1 mL solution/mg of sample) for four hours in order to remove exogenous carbonates. Samples were then rinsed five times, centrifuged and freeze-dried. Stable carbon and oxygen isotope analyses were performed at the Leibniz-Laboratory for Radiometric Dating and Isotope Research at the University of Kiel. Treated samples were analyzed using a Kiel IV carbonate preparation device interfaced with a ThermoScientific MAT 253 stable isotope mass spectrometer. Stable oxygen and carbon isotopic results are reported in per mil and were calibrated relative to the VPDB scale using NBS19 (δ^13^C = +1.95‰; δ^18^O = −2.20‰). Analytical precision was controlled based on two internal tooth enamel standards (CM1 = modern sheep tooth enamel and ER1 = archaeological cattle enamel). A total of 12 measurements from each standard from six different measurement days resulted in a SD (1σ) of 0.1‰ (CM1) and 0.05‰ (ER1) for δ^13^C and 0.15‰ (CM1) and 0.07‰ (ER1) for δ^18^O. The mean standard deviation of replicate analyses of sample duplicates (n = 4) was 0.03‰ for both δ^13^C and δ^18^O.

In order to assess hypothesized shifts in Xiongnu mobility over time, bone collagen from interred individuals was directly dated by AMS. One date was obtained from a cattle bone specimen interred in a grave and, in one case, dating was conducted on the coffin planks made from larch (S1 Table). Contexts with more than one AMS date are represented by the pooled mean radiocarbon age of all dated samples. In order to make a time line for studying changes in mobility, each radiocarbon assay was calibrated using OxCal 4.4 (IntCal 20 calibration curve) and a mean radiocarbon year was calculated as a single point estimator. This was done with full understanding that the calibrated mean of radiocarbon determination does not reflect its probability distribution [87] but does provide an approximate diachronic scaffolding that estimates the relative chronological order of distinct events such as burial interments.

## Results

### Bioavailable strontium isotope values for BGC

Tooth enamel from modern domesticated sheep that grazed in open steppe surrounding BGC underpinned by Permian sedimentary rock exhibited ^87^Sr/^86^Sr values between 0.7092 to 0.7094 and provide a local reference data set characterizing the strontium isotopic composition of lower valley pastures within BGC and desert-steppe pastures beyond the granite ridge formation (Table 1). Iron Age sheep from Xiongnu burials yielded similar values ranging from 0.7091 to 0.7093 suggesting Iron Age animals were herded locally in the BGC area. Based on the mean and standard deviation (2σ) of these modern and archaeological sheep, we define the range of bioavailable strontium isotopes local to BGC as 0.7092 ± 0.0003 (2σ).

**Table 1:** Strontium isotope values (^87^Sr/^86^Sr) measured from the tooth enamel of modern domesticated sheep (*Ovis aries)*, wild sheep (*Ovis ammon*), and archaeological sheep recovered from Xiongnu burials establishing local bioavailable strontium isotope values. M/2 = second mandibular molar.

Taxon	Sample ID	Provenance	Sampled tooth	^87^Sr/^86^Sr	2σ	Sr (ppm)	Original identifier
*Ovis aries*	4708	Modern	M/2	0.70941	0.00002	628	BGC M/2
*Ovis aries*	4709	Modern	M/2	0.70918	0.00002	331	BGC D/1
*Ovis aries*	4710	Modern	M/2	0.70936	0.00002	685	BGC R/3
*Ovis aries*	4702	Xiongnu	M/2	0.70932	0.00002	510	Ex 04.01 (BGC 576)
*Ovis aries*	4703	Xiongnu	M/2	0.70915	0.00002	631	Ex 04.01 (BGC 576)
*Ovis aries*	4704	Xiongnu	M/2	0.70906	0.00002	421	EX 03.03 (BGC 128; BGC-AR-17)
*Ovis aries*	4705	Xiongnu	M/2	0.70920	0.00002	298	Ex03.03 (BGC 128; BGC-AR-1/6)
*Ovis ammon*	4706	Modern	M/2	0.71093	0.00002	376	BGC WS 12
*Ovis ammon*	4707	Modern	M/2	0.71117	0.00002	215	BGC WS 13/3

Modern wild argali sheep local to BGC produced more radiogenic ^87^Sr/^86^Sr values of 0.7109 and 0.7112 reflecting preferential intake of graze and browse located in the granite ridges and rocky plateaus dissecting BGC. The wild argali sheep values were not used to estimate the local bioavailable strontium isotope range of BGC as it is highly unlikely that the limited distribution of more radiogenic strontium isotopes in BGC would have entered local pastoralist foodchains. While floral biomass supported on the granite ridges is sufficient for argali and ibex herds, it is inadequate in quantity and quality for sustained grazing of domesticated livestock. In addition, thin underdeveloped soils present in the plateaus and granite rocks favored by wild sheep and goats would not have supported cultivation plots, although it remains to be seen if local cultivation of millet, wheat, and/or barley took place in BGC during the Xiongnu period.

### Strontium and oxygen isotopes of human tooth enamel

**Fig 5:**
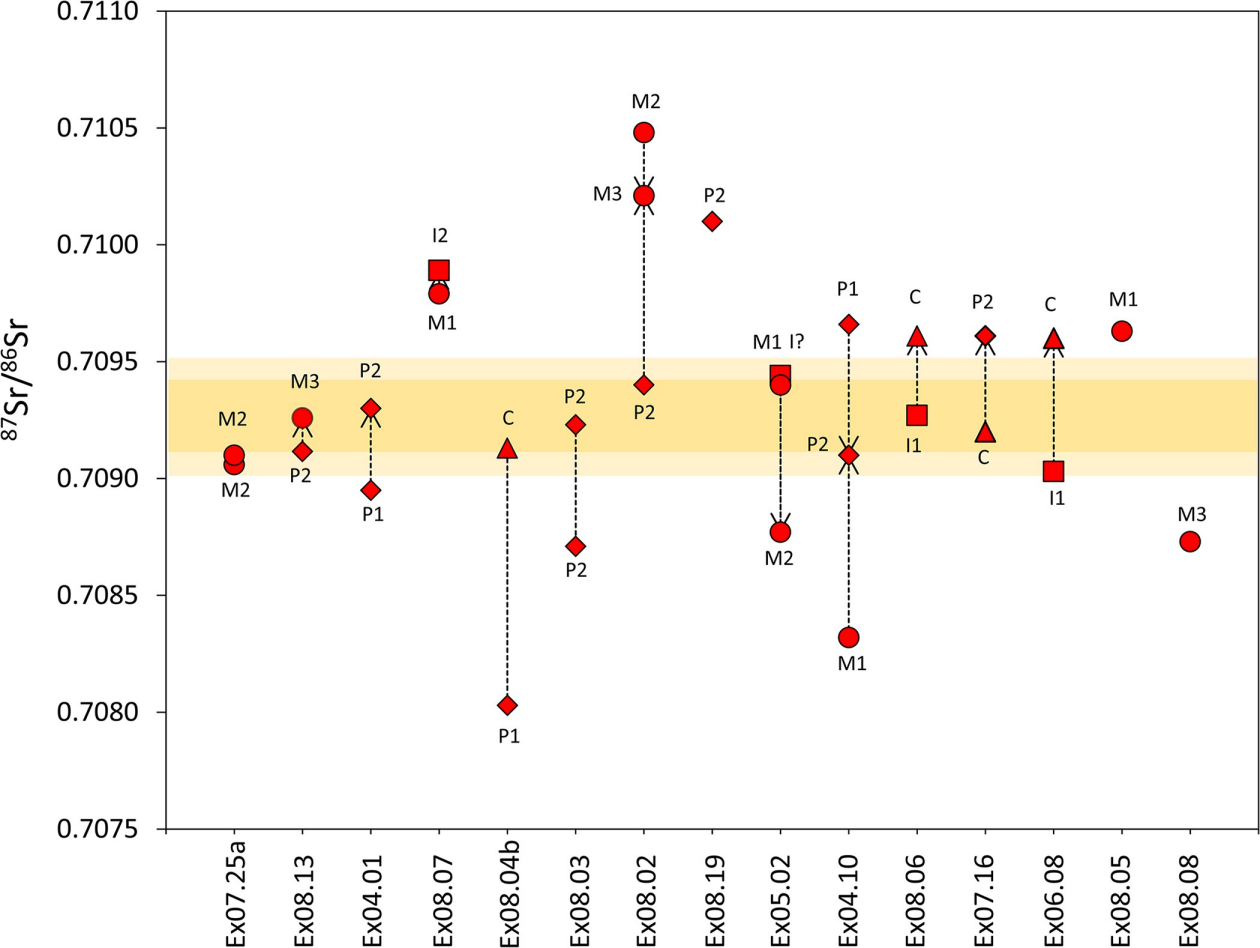
Strontium isotope values for single and paired teeth for Xiongnu individuals from Baga Gazaryn Chuluu. Yellow bars indicate range of local bioavailable strontium isotope values (light yellow = 2*σ*) measured from modern sheep grazing in BGC and sheep interred in Xiongnu graves. For paired teeth from the same individual, the arrow indicates the later forming tooth. Red shapes indicate tooth type (circle = molar, diamond = premolar, square = incisor, triangle = canine).

Strontium isotope values falling both within and outside the locally defined range for bioavailable strontium for BGC [i.e., 0.7092 ± 0.0003 (2*σ*)] reveal diverse mobility histories of Xiongnu intermediate elites. The strontium isotope ratios of Xiongnu human tooth specimens ranged from 0.7080 to 0.7105 (Table 2 and Fig 5 and S1 Table). Although not the focus of this study, we report here strontium isotope values measured for individuals recovered from a Bronze Age prone burial (Ex08.21, first molar, 0.7075), and undated cave burial likely medieval or historical in origin (Ex08.31; first molar, 0.7079), and an Early Iron Age individual from a non-standard burial feature (Ex07.19b; second premolar, 0.7094). These non-Xiongnu strontium isotope values are not displayed in Fig 5.

**Table 2:** Strontium (^87^Sr/^86^Sr), and carbon (δ^13^C) and oxygen (δ^18^O) isotope values measured from Xiongnu intermediate elites buried at BGC as well as calibrated radiocarbon determinations. Additional information reported in S1 Table.

Site	Sample ID	Element	^87^Sr/^86^Sr	δ^18^O_VPDB_ (‰)	δ^13^C_VPDB_ (‰)	Calibrated date
Ex07.25a	5004	Right M^2^	0.7091	–8.1	–11.7	380–197 BC
	Machicek et al. 2019	M_2_	0.7091	n/a	n/a	
Ex08.13	5017	Left P^2^	0.7091	–9.2	–10.3	373–180 BC
	5018	Right M^3^	0.7093	–9.8	–10.0	
Ex04.01	4996	Left P_1_	0.7090	–11.2	–11.3	390–206 BC
	Machicek et al. 2019	P_2_	0.7093	n/a	n/a	
Ex08.07	5014	Right M^1^	0.7098	–11.5	–-6.3	197–48 BC
	5015	Left I^2^	0.7099	–9.1	–10.6	
Ex08.04b	5009	Left P^1^	0.7080	–9.7	–11.5	175–8 BC
	5010	Right upper C	0.7091	–8.0	–11.0	
Ex08.03	5007	Right P^2^	0.7092	–12.1	–10.3	165–2 BC
	5008	Left P^2^	0.7087	–11.9	–10.9	
Ex08.02	5005	Right M^3^	0.7102	–11.2	–10.9	172 BC—8 CE
	5006	Left M^2^	0.7105	–10.9	–10.8	
	Machicek et al. 2019	P_2_	0.7094	n/a	n/a	
Ex08.19	Machicek et al. 2019	P_2_	0.7101	n/a	n/a	159 BC—4 CE
Ex05.02	4999	Right I (?)	0.7094	–12.8	–8.7	162 BC—7 CE
	5000	Left M_2_	0.7088	–10.5	–9.7	
	Machicek et al. 2019	M_1_	0.7094	n/a	n/a	
Ex04.10	4997	Left P_1_	0.7097	–7.4	–11.1	148 BC—21 CE
	4998	Left M^1 (?)^	0.7083	–8.1	–10.4	
	Machicek et al. 2019	P^2^	0.7091	n/a	n/a	
Ex08.06	5012	Right I_1_	0.7093	–7.8	–8.6	151 BC—62 CE
	5013	Right lower C	0.7096	–8.9	–7.5	
Ex07.16	5002	C	0.7092	–9.8	–11.1	151 BC—77 CE
	Machicek et al. 2019	P_2_	0.7096	n/a	n/a	
Ex06.08	5001	I^1^	0.7090	–8.6	–13.5	41 BC—109 CE
	Machicek et al. 2019	Lower C	0.7096	n/a	n/a	
Ex08.05	5011	Left M^1^	0.7096	–6.7	–12.9	8 CE—123 CE
Ex08.08	5016	Left M^3^	0.7087	–10.1	–8.7	42 BC—130 CE
Ex08.21	5019	M1	0.7076	n/a	n/a	Bronze Age
Ex08.31	5020	M1	0.7079	n/a	n/a	Medieval/Historical?
Ex07.19b	5003	P2	0.7094	n/a	n/a	Early Iron Age

Given these results, Xiongnu individuals interred in BGC exhibit diverse mobility histories that included movements local and extra-local to BGC, sometimes observed in the same individual. Of the 15 individuals sampled, over half of the sampled tooth specimens from Xiongnu individuals exhibit strontium isotope values that coincide with the range of locally bioavailable strontium established for BGC (Table 1 and Fig 5). Multiple individuals also exhibit strontium isotope values outside of the range of bioavailable strontium recorded for BGC, including individuals that yielded a value similar to bioavailable strontium for BGC in one tooth as well as a value outside the BGC range in an additional tooth.

Xiongnu individuals sampled here exhibit a markedly wider range of strontium isotope values in tooth enamel compared to bone. Bone apatite from a selection of Xiongnu individuals, including some individuals included in this study, exhibits values ranging from 0.7093 to 0.7102 [88]. These bone ^87^Sr/^86^Sr values fall intermediary to those exhibited by local domesticated sheep and wild argali, suggesting that the original biogenic signal in bone was replaced by radiogenic ^87^Sr/^86^Sr taken up from the burial environment. Most Xiongnu ring burials were positioned at the interface between the rocky granite outcrop and the open steppe (Fig 4). Rainwater moving radiogenic ^87^Sr/^86^Sr down from the granite into this transitional zone would explain intermediary values in human bone bioapatite. Previous work recognized that human bone strontium isotope ratios may have been impacted by diagenetic alteration [14].

We emphasize that strontium isotope values measured from teeth represent mobility events that took place during childhood. The social context and scale represented by the childhood mobility may be quite diverse, including for example, aristocratic children sent to an elite court to be raised and trained under the watch of a central authority, or a child sent to live with distant relatives due to the loss of his or her family members. While these scenarios are conceivable, we presume that children most likely circulated in the company of their families for productive, ritual, social, or political reasons. Individuals yielding ^87^Sr/^86^Sr values outside the range of bioavailable strontium isotope values for BGC were considered extra-local to BGC during the period of enamel formation, although the potential scale of extra-locality is unknown in the absence of bioavailable strontium isotope reference data for Mongolia. Within this analytical context, individual ^87^Sr/^86^Sr values were defined as either ‘local’ or ‘non-local’, a conservative binary categorization that does not account for convergence in geological substrate and bioavailable strontium values across large geographic scales. In other words, Xiongnu individuals defined as local to BGC according to their strontium isotope composition could have conceivably originated from a more distant region that shared a similar distribution of bioavailable strontium isotope values as BGC, although a local origin is a stronger probability given the number of BGC individuals that fall into the local strontium isotope range.

**Fig 6:**
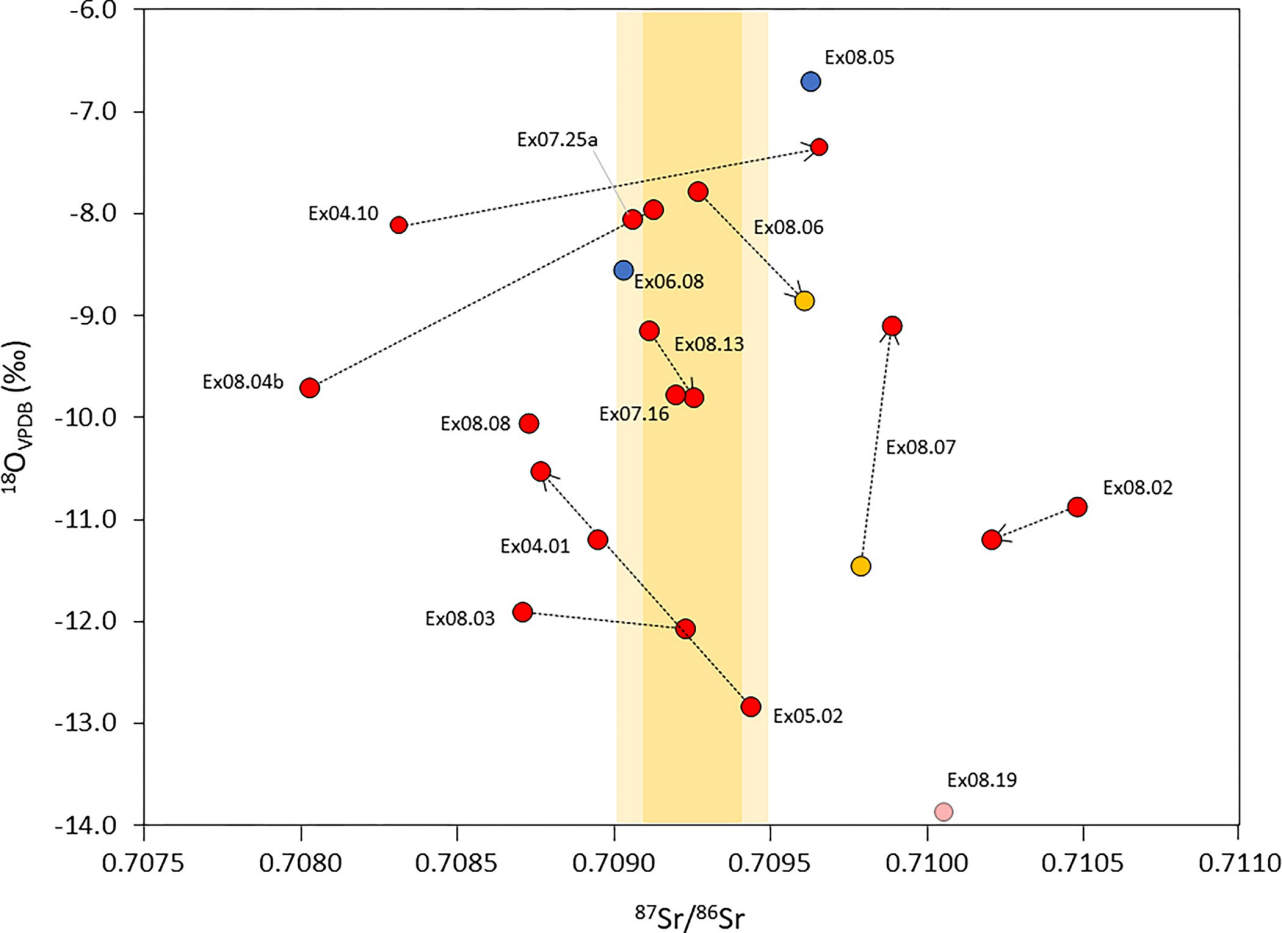
Oxygen (δ^18^O_apa_) and strontium isotope values for BGC Xiongnu individuals. Blue circles indicate individuals yielding carbon isotope values consistent with dietary intake from a C_3_ environment and yellow circles indicating individuals yielding high δ^13^C values consistent with millet consumption in a C_3_/C_4_ environment (see Fig 8). Ex08.19 was measured only for strontium isotopes. For paired teeth from the same individual, the arrow indicates the later forming tooth.

Teeth from Xiongnu period humans measured for oxygen isotopes as part of this study average –9.7 ± 1.6 in δ^18^O and exhibit a wide range of oxygen isotope values between –6.7‰ to –12.8‰ (Fig 6). Comparison of δ^18^O values of teeth with different formation times from the same individual demonstrates in general minimal oxygen isotopic differences with a 1.3‰ ± 0.9 offset on average in δ^18^O values between teeth irrespective of tooth type (Table 2). Inter-tooth oxygen isotope variation within single individuals ranges between 0.2‰ and 2.4‰. Higher δ^18^O values would be expected in portions of the earliest forming teeth when children are suckling due to ingestion of ^18^O-enriched breast milk [89]. The lack of systematic ^18^O enrichment in early forming teeth in single individuals is due to a bulk sampling strategy that samples the entire tooth grown, mixing pre-weaning and post-weaning oxygen isotope signals. There are no significant differences between δ^18^O values exhibited by individual yielding ^87^Sr/^86^Sr values similar to bioavailable strontium isotopes values for BGC (–7.8 to –12.8‰ in δ^18^O) and individuals yielding more or less radiogenic strontium isotope values than bioavailable strontium for BGC (–6.7 to –11.9‰) (Fig 6).

**Fig 7:**
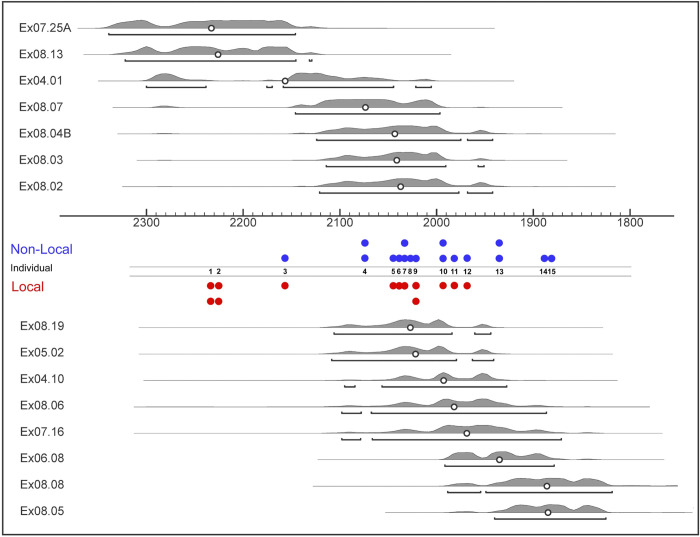
Strontium isotope results (n = 30) categorized as ‘local’ or ‘non-local’ to BGC for each Xiongnu individual (n = 15) and also displaying which individuals yielded multiple values from different teeth (see Table 2). Red or blue circles are plotted approximately in line with the mean radiocarbon year (cal BP) represented by a circle within the 95% probability distribution for the radiocarbon determination associated with each individual.

Xiongnu individuals categorized as local and non-local to the greater BGC area based on their strontium isotopic composition were plotted by mean year BP to investigate changes in locality relative to BGC over time (Fig 7). During the early period of Xiongnu state formation (late 3rd and early 2nd c. BCE), all individuals are local to the BGC area, in contrast to the early mid-second century BCE when extra-local movements begin to be regularly represented. Based on historical sources, this pattern roughly coincides with the expansion of the Xiongnu state to its full geographical extent [5]. By the late second and first century BCE, mobility was more dynamic, driven by individuals each demonstrating BGC locality mixed with BGC extra-locality (i.e., individuals yielding at least one strontium isotope value local to BGC and the other(s) non-local to BGC), suggesting these persons moved in and out of the BGC locale during childhood. Later by the first century CE, BGC elite individuals all bear an extra-local strontium isotope signature. This uptick in non-locality might have been associated with textually documented political turmoil among the ruling elite and the gradual decline of the regionally integrated polity [5].

### Carbon isotopes of human tooth enamel and Xiongnu dietary intake

**Fig 8:**
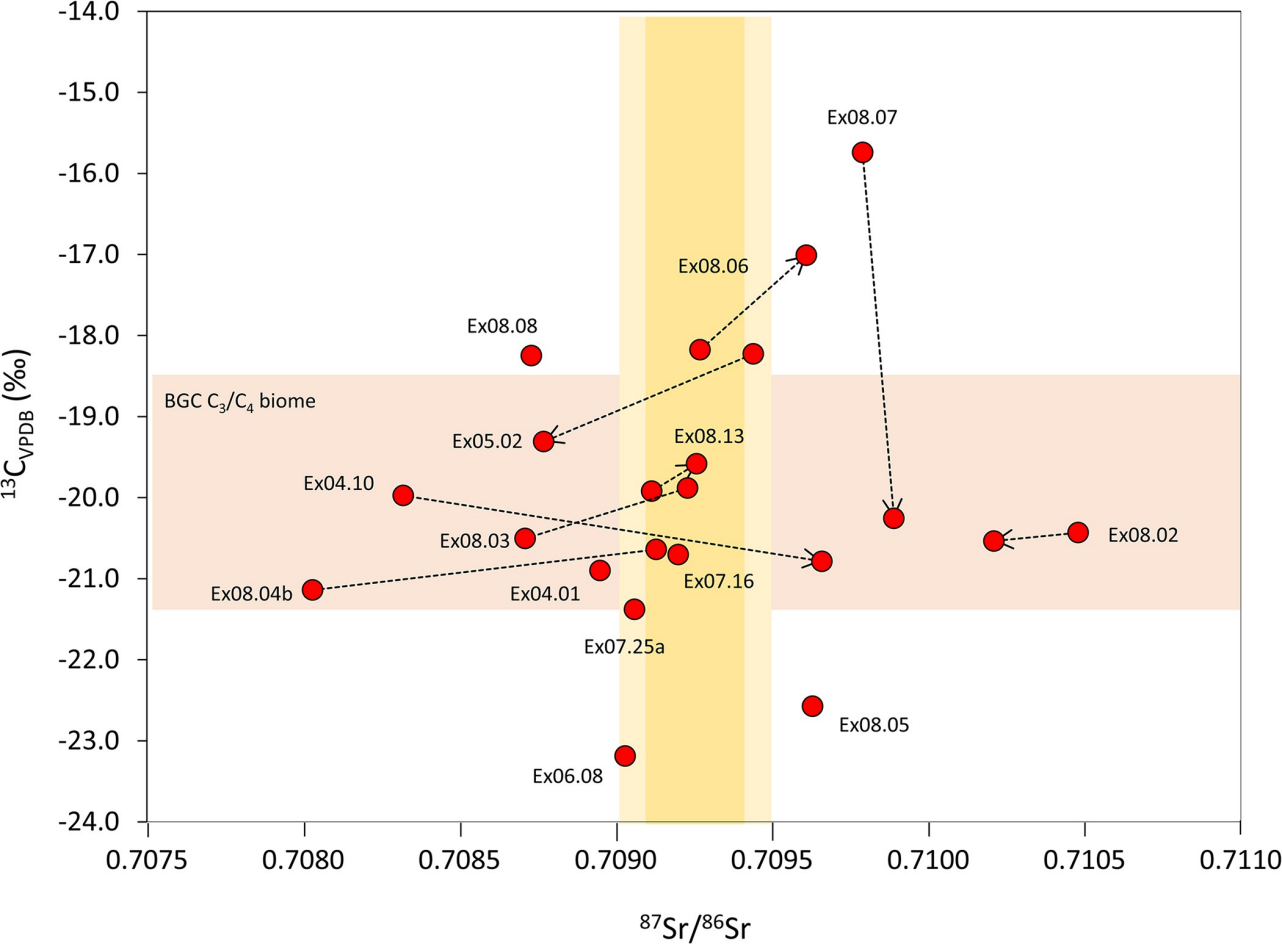
Carbon (δ^13^C_apa_) and strontium isotope values measured from Xiongnu individuals. Orange bar indicates the estimated seasonal distribution of carbon isotope values in the C_3_/C_4_ biome of the BGC area based on seasonal minimum and maximum δ^13^C_apa_ values measured from the incrementally sampled teeth of modern sheep local to BGC [62]. Yellow bars indicate range of local bioavailable strontium isotope values (light yellow = 2*σ*). For paired teeth from the same individual, the arrow indicates the later forming tooth.

Millet was variably consumed by Xiongnu elites interred in mortuary monuments situated in central Mongolia, and macro-botanical evidence for millet has been discovered in both mortuary and habitation contexts [88]. Despite this growing body of evidence for millet use, the extent to which millets were locally grown, regionally circulated, or imported from China is unknown [90]. Xiongnu individuals from BGC average −10.3‰ ± 1.6 in δ^13^C and exhibit a wide range in carbon isotope values across individuals from −13.5‰ to –6.3 (Table 2 and Fig 8). A pure C_3_ diet would yield carbonate δ^13^C values between– 20‰ to –13‰ while a pure C_4_ diet would yield values from– 4‰ to –2‰ [91]. Individuals Ex06.08 and Ex08.05 yielded the lowest values, −13.5‰ and −12.9‰, respectively, consistent with the ingestion of an almost exclusively C_3_ diet. It is likely that these individuals spent time primarily in the meadow steppe of the northern Mongolian plateau where the floral base of the foodweb is depleted in ^13^C. This is demonstrated, for example, by sheep and goats interred in Xiongnu ring burials located in Egiin Gol (northern central Mongolia) yielding low δ ^13^C_diet_ values ranging from ca –24 to –22 ‰ ([88] (5‰ diet-collagen fractionation factor according to Koch [92]).

Xiongnu elites who spent the early portion of their lives in the ^13^C-enriched environments of the Gobi steppe-desert may have consumed millet, but comparative carbon isotope data indicate firstly that the C_4_ contribution to the diets of Xiongnu individuals is not necessarily sourced solely from millet but could reasonably be derived from ingestion of pastoral products of livestock feeding in a C_3_/C_4_ biome. Domesticated sheep from contemporary herds feeding in the C_3_/C_4_ Irano-Turanian vegetation that characterizes BGC (and the broader Gobi steppe-desert) yield moderately high, seasonally directed tooth carbonate δ^13^C_diet_ values ranging from −21.6‰ ± 0.5 to −18.5‰ ± 0.6 (using a 12‰ apatite-diet fractionation factor) [62]. While one non-local individual (Ex08.07) exhibited a very high dietary δ^13^C_diet_ value in the early forming permanent first molar (−15.8‰) indicating ingestion of strongly C_4_ diet, all other Xiongnu individuals yielded more intermediate carbon isotope values ranging from −11.7‰ to −7.5‰ (δ^13^C_diet_ = −21.4‰ to −17.0‰) that resonate with the carbon isotopic composition of local livestock. The most parsimonious explanation is that intermediate carbon isotope ratios exhibited by Xiongnu individuals reflect the isotopic composition of the meat, milk, and fats of their livestock–local herd animals that grazed on C_3_/C_4_ plants. It is important to consider that millet may have been reserved for adult consumption, which may also explain the absence of high δ^13^C values in enamel tissues formed during childhood.

## Discussion

### Oxygen isotope evidence for human mobility ambiguous in steppe environments

Oxygen isotopes in tooth enamel are often measured alongside strontium isotopes in order to better resolve the geographic origins of individuals, particularly when there is low variation in the strontium isotopic composition of the sampled group. The use of human δ^18^O values to establish scales of movement has been most commonly used in temperate European environments where meteoric waters exhibit low seasonal variation in oxygen isotopes but express moderate geospatial isotopic distinction at regional scales (e.g. 5‰) [47, 93, 94]. The lack of systematic relationship between oxygen and strontium isotope values visible in Xiongnu elites from BGC is likely due to equifinality (i.e., bioavailable strontium ratios similar to those observed for BGC), but different meteoric water δ^18^O values, characterizing other regions of Mongolia, pronounced seasonality in meteoric water oxygen isotopes for the Mongolian steppe, and/or diverse oxygen isotope compositions of imbibed liquids.

Meteoric water δ^18^O values in the Mongolian steppe predictably increase with a corresponding decrease in latitude reflecting the shift from wetter, cooler environments of the northern forest-steppes to the drier and warmer climates of the desert-steppe [60, 61, 95]. The absence of a relationship between δ^18^O and strontium isotope values expressed in Xiongnu individuals suggests that the remarkably strong seasonality in Mongolian steppe meteoric water oxygen isotopes, reaching on average a low value of approximately −28‰ during the winter months and a summer high value of −9‰ [35, 36], disrupts spatial information that might otherwise be recorded in the extended, multi-year enamel formation period of the tooth crown. It may be that establishing human mobility on the basis of tooth enamel δ^18^O values may be more viable in temperate environments where there is comparatively low seasonal variation in the oxygen isotopic composition of meteoric waters, geospatial consilience between meteoric water, groundwater, and riverine δ^18^O values, and high humidity that reduces evaporative ^18^O enrichment of open water sources. Even so, the high degree of overlap in human δ^18^O values from different locations across temperate European environments strongly cautions against using oxygen isotopes alone to assess precise geographic origins of individuals or identify non-locals when sample sizes are small (< 25 individuals) [84].

People imbibing water sources of isotopically diverse origins would also explain the wide oxygen isotopic variation. Despite predictable geospatial variation in the oxygen isotopes of meteoric water across Mongolia [96], the oxygen isotopic compositions of open water sources across the steppe do not necessarily exactly reflect the roughly northwest to southeast gradient from low δ^18^O values to high δ ^18^O values seen in meteoric water oxygen isotope ratios. Large, deep lakes in Mongolia exhibit oxygen isotope ratios that broadly track meteoric δ^18^O values [60, 97], but the oxygen isotopic composition of water held in small lakes deviates sharply from expected local meteoric water δ^18^O values due to evaporative enrichment processes that are particularly pronounced in semi-arid Mongolian environments. The oxygen isotope ratios of seasonal shallow lakes are strongly influenced by depth and exhibit a wide range in δ^18^O values even on local scales within the Mongolian steppe [97]. Although Xiongnu pastoralists probably preferentially obtained their drinking water from deep lakes and rivers, springs, and perhaps wells, shallow ^18^O-enriched seasonal lakes and ponds were likely also accessed, especially in the Gobi steppe-desert where permanent water sources are rare.

Culturally mediated drinking practices would also further interrupt spatially sensitive δ^18^O inputs from meteoric water and provide another source of variation in enamel carbonate δ^18^O values. Imbibing drinking water stored in containers or charged from sources other than those directly derived from local meteoric precipitation (e.g., rivers or wells in some regions) [98], food preparation practices such as boiling and brewing that alter the oxygen isotopic composition of water [99], or sustained consumption of ^18^O-enriched milk would likely further influence the oxygen isotopic composition of bioapatite δ^18^O [100, 101]. Food and beverage preparation techniques for the Xiongnu period are unknown but likely included ^18^O-enriched milk and teas widely consumed by Mongolian pastoralists today. Breastfeeding imparts an approximately 0.5–0.7‰ enrichment in tooth enamel ^18^O due to preferential incorporation of ^18^O isotopes into body water [89, 102]. After weaning, oxygen isotope ratios in calcified tissues decrease to reflect the oxygen isotopic composition of local meteoric water [103]. While the permanent dentition sampled from BGC elites were formed after weaning, the ingestion of livestock milk–a major source of dietary intake in pastoralist societies–may also similarly alter the oxygen isotopic composition of human bioapatite.

### Local pastoral production a mainstay for Xiongnu commoners and elites?

Our sample of archaeological herd animals is limited to four individual sheep recovered from two Xiongnu period mortuary contexts. Although not a robust sample, strontium isotopic identity between contemporary BGC sheep known to have remained local to BGC and Xiongnu period sheep suggests Xiongnu herding was concentrated in the BGC area. This interpretation is consistent with nitrogen isotopic evidence for the repetitive seasonal placement of livestock on winter grazing grounds indicating established pastoralist infrastructure and, likely, associated pasture rights during the Xiongnu period [12]. Taken together, these data indicate that some sectors of the pastoral economy likely remained spatially limited, focused on household subsistence and small-scale wealth generation rather than on expansive pastoral production associated with the generation of staple finance.

However, a few caveats should be considered when interpreting these patterns. First, our relatively small sample may have missed greater variation in Xiongnu herding practices and especially those related to periods of resource downturn associated with severe droughts and cold snaps. Ethnographic evidence suggests that during times of local hardship, movement constitutes a primary strategy to secure adequate graze and water for herd animals in need [104]. Ethnographic interviews of wealthier BGC pastoralists with substantial herds reveal that movement regimes reaching as far as 80 to 100 km beyond the BGC area, notably northwards to the Bayan Onjuul region, are today not uncommon (Fig 3). However, the number of BGC herders that can support such long range movement is relatively few. Wealth in herds was probably one characteristic of local elite lifestyles during the Xiongnu period, but we cannot assume that animals found in burial contexts represent herds owned by, herded by, or tied to the interred elite individual or his or her household. Interred herd animals were probably consumed as part of funerary feasts and could have been supplied for that purpose by commoners, funeral participants, or other local elite groups. As such, the degree of indeterminacy associated with mortuary contexts makes it difficult to generalize about local pastoralist strategies at BGC.

There may have been ample opportunities for expanded pastoral ranges and freedom of movement bolstered by political consolidation within the Xiongnu polity, but such enabled mobility may have benefitted common herders more so than elite and may have been practiced mainly during times of urgent subsistence need [6]. While mobility decisions related to pastoralist subsistence and surplus production are influenced by a wide range of factors (e.g., pasture availability, grazing rights, and distribution of other pastoralist infrastructure) [105], non-pastoral subsistence pursuits, activities such as craft manufacture, as well as socio-political concerns further shaped the timing, direction, and scale of movements [20]. In all likelihood, Xiongnu period herding involved a combination of flexibly scaled movements, some shorter- and some longer range, that were put into practice as needed by knowledgeable and skilled local herders. Additional strontium isotope analyses of Xiongnu cattle, sheep, and goats would help further resolve the geospatial scale of livestock herding in the BGC area.

### Regional Xiongnu political consolidation embedded in translocality

The correspondence between local bioavailable strontium isotope values and the ^87^Sr/^86^Sr ratios of many Xiongnu individuals, along with their eventual burial at BGC, argues for lasting ties to this local area. However, visible in the strontium isotope ratios and also carbon isotope values of several individuals is a clear pattern of in-and-out movement suggestive of extra-local circulation beyond BGC. This pattern is best thought of as socio-spatial tethering among some BGC elite households, presumably moving with their children, a practice that became common sometime during the late second to the late first century BCE (Fig 7). This circulation of local elites within an expanded regional space probably fostered network and alliance building in support of new enlarged forms of power-holding factions. This suggests one path towards the formation of a geographically centered aristocratic gentry. These may have included aristocratic houses that were principle actors in the negotiated politics that underwrote large-scale nomadic states [7].

Bringing together various lines of evidence including regional archaeology patterns, historical accounts, and research at other local areas, our analyses of BGC mobility foster hypothesis building for the ways that a nomadic state may have gradually come together. We propose that local leadership likely achieved their position through their own efforts to engage spatially dispersed mobile households within a regional home territory and garnered political support through their own movement and circulation. This would have enabled opportunities for local leaders to emerge as crucial ‘go-betweens’ negotiating relationships that articulated local political sentiment with regional political conditions and vice versa. In other words, local power holders had the opportunity to become power brokers (i.e., nascent intermediate elites) as the process of state formation was underway. These emergent elites communicated regional exigencies of local mobile pastoralists to regional-level leadership whose power and authority were partly dependent on support from local leaders. The consistent ancillary presence of ‘non-local’ intermediate elites in BGC further attests to the translocal nature of Xiongnu socio-political structures.

That some of the individuals interred at BGC clearly spent their childhood in locales at a distance from their home territory speaks to the importance of translocality and movement in nomadic polities. These externally experienced persons, who as adults would have been privy to discrete sets of political knowledge unfamiliar to everyday herders and elites who had remained local to BGC, were conduits through which information flowed and external relations would have been established, linking BGC into a network of political affiliates. These persons could have further parlayed such information and their positions to build, consolidate, and breakdown political power within these factions to create alternative networks and modes of authority. That external persons appear to have originated from multiple places across the Xiongnu polity, rather than emanating from a single place, and then subsequently integrating with a core cadre of Xiongnu elites who had remained in BGC throughout their life, is important. This speaks not only to the deep-seated stability of regional political factions but also to established multi-placed translocality within the Xiongnu polity relations, a configuration enabled by a high degree of socio-political and cultural embeddedness in both the place of origin and the place of destination. Accordingly, these data are suggestive of dual processes of state making under way during this period. As the Xiongnu state expanded territorially to the east and west by way of both conquest and negotiation [4], centrally located political actors consolidated and formalized their own intermediate level factions to both balance and benefit from the expanding power exercised by the central court of the Xiongnu.

### Conclusion

The hypotheses offered here require testing at other local areas in Mongolia and at the regional scale of archaeological analysis as well. Based on our analyses, we find that movement and politics were thickly intertwined in the Xiongnu steppe polity. Mobility expanded opportunities for intra- and inter-community interaction whether through formal, scheduled events such as monument building or informal everyday practices involving livestock herding. Xiongnu intermediate elites, such as those who were eventually memorialized at BGC, participated in different scales of movement ranging from local circulation, in-and-out movements, and trans-regional relocation. These varied modes of mobility enabled the formation of consolidated regional power factions that were then linked to more distant factions within the Xiongnu polity. The outcome of these political contacts produced a robust and flexible power-sharing structure well suited to mobile communities scattered across the vast distances of the steppe. First, sustained locality by a core of intermediate elites promoted intra-regional network building and alliance formation, the principle constituents of state formation. Second, the consistent presence at Baga Gazaryn Chuluu of extra-local intermediate elites, purveyors of information and political knowledge unfamiliar to local intermediate elites and common herders, suggests that trans-regional mobility integrated far-flung political factions to effectively bind together the Xiongnu state.

From a comparative and anthropological point of view, our research points to novel ways for understanding ancient statehood and processes of political complexity. Although the pastoral nomadic states and empires of Mongolia and other parts of Eurasia are fascinating topics unto themselves, their unique statecraft, diverse citizenries, and expansive scales point us in promising directions for reconceiving the nature of statehood. The typical conception of an ancient state as sedentary, urban, administrative, and dependent on intensive agriculture presents a limited model for understanding the nature of political processes that comprised early states. Disrupting this long-standing conception of statehood is the decidedly atypical case study of the Xiongnu state–a polity that was extremely large in scale, socially and politically stratified, non-urban, highly mobile and expert in long-distance networking, and militarily powerful. While the management of nucleated, surplus-producing populations was a problem addressed by sedentary statecraft, the nomadic states of Eurasia innovated methods for maintaining state ideologies, behavioral codes, and resource acquisition over tremendous areas with dispersed nomadic populations. In the case of the Xiongnu state, new forms of regional organization were devised emphasizing a mobile, spatially informed, and more flexible brand of politics that was to become a long-standing tradition on the eastern Eurasian steppe.

## Supporting information

S1 Table Multi-stable isotope measurements, radiocarbon determinations, and osteological data for Xiongnu intermediate elites from Baga Gazaryn Chuluu. (XLSX)
